# Fetus in the Bladder: Rare Complication of Vesicouterine Fistula

**DOI:** 10.1155/2016/5750710

**Published:** 2016-12-26

**Authors:** Vladimir Lesovoy, Yuryi Parashchuk, Dmytro Shchukin, Roman Safonov, Karyna Didenkova, Maria Lisova, Inessa Safonova

**Affiliations:** ^1^Kharkiv National Medical University, 4 Nauky Avenue, Kharkiv 61022, Ukraine; ^2^V.I. Shapoval Regional Clinical Center of Urology and Nephrology, 195 Moskovskyy Avenue, Kharkiv 61037, Ukraine; ^3^Regional Clinical Hospital, Center of Emergency Medical Care and Disaster Medicine, 13 Pravdy Avenue, Kharkiv 61022, Ukraine; ^4^Kharkiv Medical Academy of Postgraduate Education, 58 Amosova Street, Kharkiv 61176, Ukraine

## Abstract

The work presents a rare case of spontaneous migration of an 11-week fetus from the uterine cavity into the urinary bladder cavity through the long-standing vesicouterine fistula.

## 1. Introduction

Vesicouterine fistula is an abnormal passageway between the cavities of the bladder and uterus. This condition is very rare and accounts for 1% to 4% of all urogenital fistulas, including vesicovaginal fistula, ureterovaginal fistula, and urethrovaginal fistula [1, 2]. The most common causes of this problem are considered to be iatrogenic injuries resulting from Cesarean section, use of vaginal forceps, as well as cervical dilation and uterine cavity curettage abortion [3–5]. The more rare causes of this type of fistulas include embolization of uterine arteries, vaginal birth after previous Cesarean section, migration of intrauterine devices, placenta percreta, and endometriosis. The literature also describes anecdotal cases of spontaneous development of vesicouterine fistulas, as well as cases of their formation as a result of necrosis of uterine fibroids, chronic inflammation, or radiotherapy [6–8].

Clinical presentations of vesicouterine fistulas may be early or late and are mostly represented by amenorrhea, urinary incontinence, and macroscopic hematuria during menstruation (menouria) [2, 9].

Although there are reports in the literature about pregnancy in the presence of vesicouterine fistula, cases of movement of the fetus through the fistulous tract are extremely rare [10–12]. We present a survey of spontaneous migration of an 11-week fetus from the uterine cavity into the urinary bladder through the long-term vesicouterine fistula.

## 2. Case Report

Patient K., a 39-year-old female was admitted to a general hospital in Severodonetsk (Ukraine) on 14 December 2015 with complaints of severe lower abdominal pain radiating to the lumbar region, as well as difficulty urinating.

She had a history of Cesarean sections in 2003 and 2005, respectively. The latest Cesarean section ended up with iatrogenic injury to the apex of the bladder. The defects of the bladder and uterus were repaired separately. The patient was in satisfactory condition when discharged from the department of obstetrics.

However, after three months the patient developed severe abdominal pain at the end of urination associated with scanty periods accompanied by hematuria. On the basis of characteristic symptoms vesicouterine fistula was suspected. This diagnosis was confirmed by cystoscopy. The patient was offered surgery, which she refused. Subsequently, due to resolution of symptoms the patient stopped seeking medical attention, although she periodically suffered from urinary incontinence, menouria, and recurrent infection of lower urinary tract.

**Figure 1:**
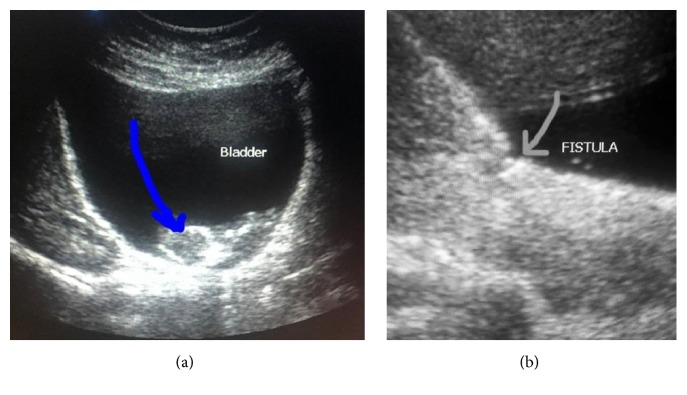
Ultrasound images demonstrate (a) a fetus in the mother's bladder and (b) the fistulous tract between the cavities of the uterus and bladder.

After examination the patient was diagnosed with missed abortion of ectopic pregnancy at 11 weeks of gestation localized in the bladder. The fetus in the cavity of the urinary bladder was detected at ultrasound examination (Figure 1). The vesicouterine fistula was also well visualized. For further examination the patient was referred to Kharkiv Regional Clinical Perinatal Center and afterwards was transferred for treatment to Regional Clinical Hospital, Center of Emergency Medical Care and Disaster Medicine, Kharkiv Regional Perinatal Center.

**Figure 2:**
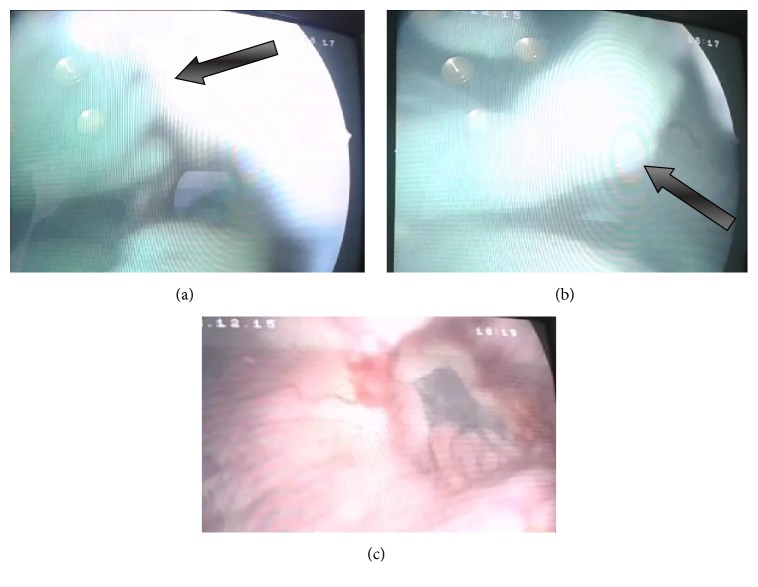
Images obtained at cystoscopy. ((a) and (b)) a dead fetus in the bladder cavity and the fetus' arms are visualized (arrows). (c) A mouth of fistula from the bladder side.

On 15 December 2015 the patient underwent cystoscopy, which revealed in the cavity of the urinary bladder a dead fetus, embryonic vesicle, and fragments of the umbilical cord (Figure 2). On the posterosuperior wall of the bladder a deck-edged mouth of fistula 2.0 cm in diameter was visualized.

On 17 December 2015 a joint team of urologists and obstetrician-gynecologists performed the following surgical treatment: laparotomy, vesicouterine fistula excision, removal of the fetus from the bladder cavity, bladder defect closure, and supravaginal amputation of uterus with removal of both fallopian tubes.

**Figure 3:**
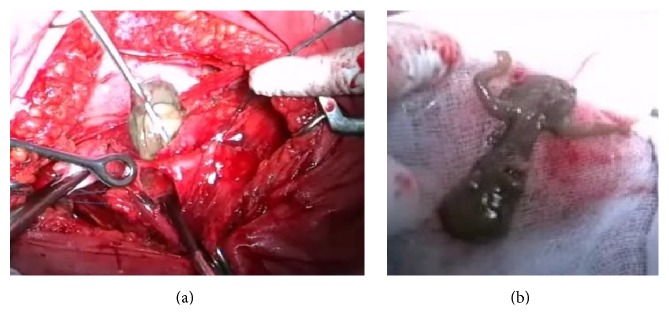
(a) The stage of fetal removal from the bladder cavity. (b) The dead fetus with signs of autolysis.

**Figure 4:**
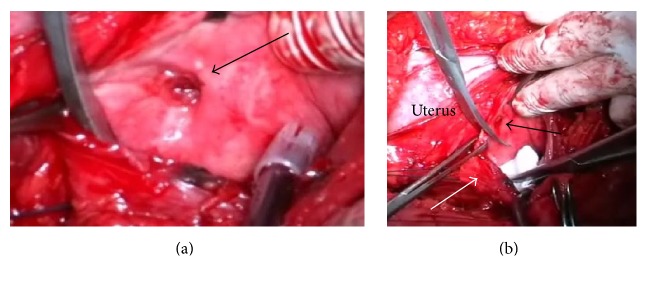
Intraoperative images. (a) View of the fistulous tract from the side of the bladder (arrow). (b) Separation of the bladder (white arrow) from the fistulous tract (black arrow).

In the course of the operation some severe adhesions were found between the bladder, uterine body, and the parietal peritoneum. The uterus measured 11.0 × 9.5 × 10.0 cm, with pale pink color, softish consistency, and smooth outer surface. The uterus had limited mobility due to adhesions. Using the method of sharp dissection the bladder was separated from the anterior surface of the uterus toward the level of the fistulous tract. The bladder was opened. The body of macerated fetus was removed from the bladder cavity along with fetal membranes with clear signs of autolysis (Figure 3). The bladder was circumferentially dissected away from the uterus around the fistulous tract (O'Connor's technique) (Figure 4). The bladder was closed with Vicryl locking sutures, and Foley catheter was inserted into the bladder cavity through the urethra. Finally, supracervical hysterectomy was performed.

The postoperative period was uneventful. Patient was discharged from the hospital at the 8th day. The urethral catheter was removed 14 days after surgery.

## 3. Discussion

Among all types of urogenital fistulas the vesicouterine fistula is considered to be the most rare one. However, there are quite a number of reports available addressing this subject [1–9]. It is established that vesicouterine fistulas occur most often in patients who previously underwent several Cesarean sections. Concerning the diagnostic aspects of this type of fistulas, it should be noted that they are accompanied by a characteristic clinical picture, including incontinence, menouria, and menstrual disorders [2, 9]. The diagnosis can be easily established by cystoscopy and roentgenologic examination (cystography, hysterography), as well as by modern methods of visualization (ultrasonography, MDCT, or MRI).

Scientific publications have presented some clinical cases of pregnancy accompanied by vesicouterine fistula; however, only three cases of embryonic or fetal migration into the bladder cavity have been reported so far. Guruvare S. and coauthors reported a case of a patient with macrohematuria and blood clots in the bladder, who had undergone Cesarean section 18 months earlier [10]. At ultrasonography a large mass in the bladder was suspected, though histological examination revealed a blood clot, which contained fragments of an embryo.

The first case of finding a fetus in the bladder was reported by Banale and coauthors [11]. A patient, who previously had had two Cesarean sections, presented with complaints of pain in the abdomen and macrohematuria. A day before she had undergone an abortion at 17 weeks' gestation. Ultrasound examination revealed a fetus in the bladder cavity. Evidently, dilatation of the cervical canal or curettage of the uterine cavity caused damage to the anterior uterine wall and the posterior bladder wall, allowing the fetus to migrate into the bladder. At cystoscopy a dead fetus and a mouth of a fistula 2.0 cm in diameter on the rear wall of the bladder was discovered. The patient underwent removal of the fetus from the bladder with separate repair of defects of the bladder and uterus.

Another publication by Gupta and coauthors presented a report about prolapse of amniotic sac through a vesicouterine fistula at 22 weeks of pregnancy [12]. The patient had history of two Cesarean sections. The main clinical presentations were severe pain and difficulty urinating. Examination revealed that the amniotic sac prolapsed through the urethra and could be displaced by finger or when placing the patient in Trendelenburg position. Cesarean section and vesicouterine fistula suturing were performed.

Our clinical observation is unique in several ways. In particular, beginning of the pregnancy was presented along with the existence of vesicouterine fistula, which had developed more than 10 years earlier. In contrast to the observation by Banale and coauthors, in our case we registered migration of quite a large fetus (11 weeks) through the long-term fistulous tract with diameter of approximately 2.0 cm. Apparently, the fistula size increased as long as the size of the uterus was increasing, which enabled the fetus to move into the bladder. The exact time of the fetus migration was undetectable since the clinical picture had developed 3 days before the surgery; however, the morphologic changes conformed to the longer term of death of the fetus. Probably, the fetus had been already dead by the time of its migration, and after its transfer the fistulous tract significantly decreased.

It should be noted that our patient had a history of two Cesarean sections. The clinical symptoms of vesicouterine fistula had not seriously bothered the patient until the admission to the hospital, and 11 weeks of pregnancy had passed without any complications. Although the existing surgical methods applied to correct vesicouterine fistula involve both preservation and removal of the uterus, we utilized the non-organ-sparing option due to the age of the patient and according to her decision.

For this patient the O'Connor's technique was used [13]. The procedure involves a sagittal cystotomy (bivalving the bladder) or posterior cystotomy that is carried toward the edge of the fistula. The fistula tract is excised and the bladder defect is closed with a running suture. In our opinion, the technique is the most convenient in the patients with vesicouterine fistulas.

## 4. Conclusions

The onset of pregnancy is possible in the presence of the long-term vesicouterine fistula. The migration of the fetus from the uterine cavity into the bladder presents one of the complications of vesicouterine fistulas.
